# Quality of Life and Clinical Outcomes in Patients with Skull Base Chordoma and Chondrosarcoma Treated with Pencil-Beam Scanning Proton Therapy

**DOI:** 10.3390/cancers17223651

**Published:** 2025-11-13

**Authors:** Katarina Bryjova, Paul-Henry Mackeprang, Dominic Leiser, Damien C. Weber

**Affiliations:** 1 Center for Proton Therapy, Paul Scherrer Institute, ETH Domain, CH-5232 Villigen, Switzerlanddominic.leiser@psi.ch (D.L.); 2 Department of Radiation Oncology, Inselspital, Bern University Hospital, University of Bern, CH-3011 Bern, Switzerland

**Keywords:** proton therapy, skull base tumors, chordoma, chondrosarcoma, quality of life, QoL

## Abstract

**Background**: skull base chordoma (Ch) and chondrosarcoma (ChSa) are rare neoplasms prone to local relapse. Alongside surgery, proton therapy (PT) is a well-established treatment for them. Given the relatively long patient survival expectancy, post-treatment quality of life (QoL) is crucial. This study prospectively assessed long-term QoL in this collective. **Methods**: seventy-seven adult patients (median age, 50 years; male *n* = 31; 40.3%) with skull base Ch/ChSa completed at least two EORTC-QLQ-C30 and BN20 questionnaires during and after PT. Oncological outcomes and therapy-related toxicities were recorded during follow-up. QoL was analyzed, with post-treatment scores compared to each patient’s baseline and correlated to oncological outcomes. **Results**: median follow-up was 51 months (range, 1–94), with 5-year overall survival (5yOS) and local control (5yLC) rates of 88.8% and 82.8%, respectively. The time to local or distant failure ranged from 8 to 58 (median, 22) months. QoL deteriorated directly at completion of PT and two to three years thereafter, especially in patients with local or distant failure. From the fifth year onward, QoL improved again. Complete resection before PT correlated to better QoL at all time points. Disease progression was associated with overall worse QoL, higher neurological symptoms already before PT, and higher symptom burden one year thereafter. Males reported better QoL before and one year after PT than females. **Conclusions**: PT achieves excellent OS and LC in patients with skull base Ch/ChSa. QoL declines directly after PT but remains close to reference population values. From the fifth year onward, QoL improves again. Gender, resection status, and disease progression significantly affect QoL in these patients.

## Simple Summary

Understanding of long-term QoL in skull base Ch and ChSa patients following PT is essential due to their relatively long survival. Previous data on this aspect was limited to only one study with a short follow-up period. Our study aims to fill this gap by providing the first analysis of QoL in these patients in a long-term follow-up setting. We correlated the QoL data to oncological outcomes and identified three main factors affecting QoL in this population. Our findings highlight the importance of consideration of QoL in comprehensive patient care and treatment decision-making for skull base chordoma and chondrosarcoma.

## 1. Introduction

Skull base chordoma (Ch) and chondrosarcoma (ChSa) are both rare primary tumors of the bone and cartilage, with Ch having an incidence rate of <0.1 per 100,000 per year [1]. ChSa of the skull base is even rarer, accounting for approximately 0.15% of all skull base tumors [2]. Although Ch and ChSa both share histologically low-grade characteristics, they tend to be locally aggressive and to relapse locally [3]. They diverge, however, in their recurrence rates and prognosis, with ChSa exhibiting a more favorable outcome after surgery and radiotherapy [4].

The challenging nature of these tumors arises from their predominantly unfavorable localization in close proximity to the optic apparatus (OA), brainstem, or neurovascular structures. While surgical resection is an effective treatment for Ch and ChSa, achieving a complete resection is rarely feasible due to difficulties associated with the aforementioned anatomical structures, which are in the direct vicinity of the tumor. Next to surgery, irradiation is a well-established treatment modality for these tumors. Providing a high dose conformality and thus delivering a high dose to the target volume while sparing the surrounding healthy tissue, proton therapy (PT) is very well suited for brain and skull base tumors [5]. Previous PT series of skull base Ch and ChSa have shown favorable long-term outcomes, with consistent differences between histologies. Hug et al. reported 5-year local control (LC) of ~70% and progression-free survival (PFS) of 65–75% for Ch, compared to LC > 85% and PFS > 80% for ChSa, with overall survival (OS) ranging from 75 to 85% [6]. Later spot-scanning series confirmed similar 5-year LC (60–75%) and PFS (55–70%) for Ch versus LC of 80–90% and PFS > 80% for ChSa [3]. Reviews of larger cohorts report 5-year OS commonly within 70–90%, again favoring ChSa [7,8,9,10]. While many of these studies demonstrated the efficacy and safety of PT in skull base Ch and ChSa [6,7,11], patients’ post-treatment QoL remained largely unexplored. One singular study by Srivastava et al., 2013 [12] examined the QoL in Ch and ChSa patients during and immediately after PT. Longer follow-up data, however, were not reported. Considering the anticipated rather long survival of these patients, with their well-being at the center of the interest, it is essential to understand the impact of treatment on their QoL beyond the therapeutic period. This study aims to provide insights into the long-term QoL of patients with skull base Ch and ChSa following pencil-beam scanning PT.

## 2. Materials and Methods

### 2.1. Study Design and Population

**Figure 1:**
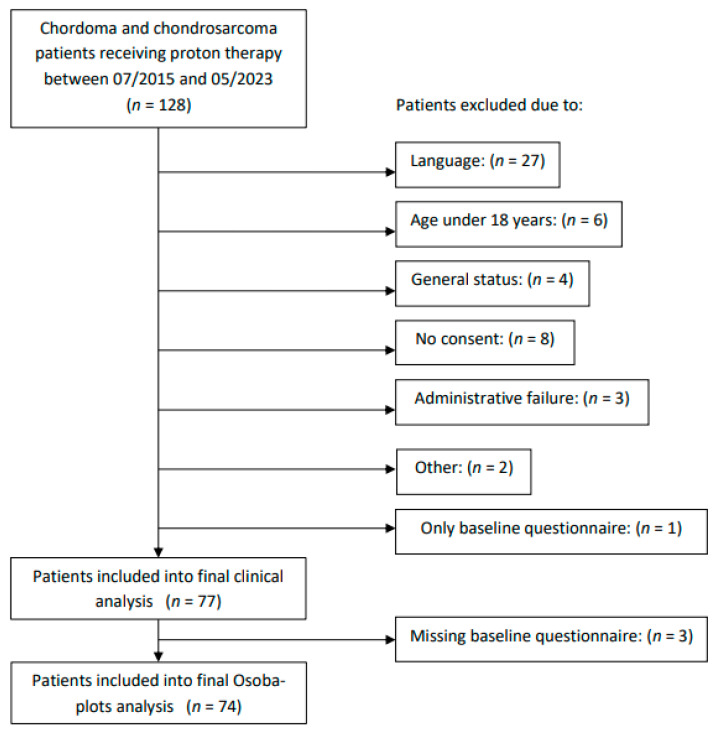
Flow diagram of the exclusion process.

Eligible patients were ≥18 years of age and were treated for skull base Ch or ChSa between July 2015 and May 2023. PT was delivered with curative intent in the adjuvant setting after gross total or subtotal resection, as definitive treatment for unresectable tumors, or as salvage in selected recurrent cases. All cases were discussed and referred from a multidisciplinary tumor board. All patients had provided written consent for participation in an overarching cohort study on QoL (ethics commission approval EKNZ 2015-285). In total, 128 patients fulfilled these inclusion criteria. They were prospectively followed with European Organization for Research and Treatment of Cancer Quality of Life Questionnaire (EORTC-QLQ-C30, Version 3.0) and the Brain Neoplasm 20 Module Questionnaire (BN20) in either German, English, French, or Italian language. Patients filled in questionnaires in paper or electronic form directly before, during, at completion of PT, and annually thereafter. They were included in the analysis if at least 2 questionnaires under PT were present. In total, 77 (60%) such patients fulfilled all the study criteria (Figure 1).

### 2.2. Instruments

EORTC-QLQ-C30 and BN20 are standardized and validated questionnaires designed to assess the QoL, specific functional aspects, and symptoms in cancer patients, particularly those with brain tumors in the case of BN20 [13].

The EORTC-QLQ-C30 is composed of 30 items measuring various dimensions of health-related QoL in the week preceding assessment. Twenty-eight questions have a Likert scale with four response options (“not at all”, “a little”, “quite a bit”, and “very much”), and two questions assess global QoL utilizing a seven-point scale (from 1 = “very poor” to 7 = “excellent”). The first twenty-eight items are categorized into five functional and eight symptom scales. The two global QoL items provide an overall assessment of the patient’s QoL. The five functional scales assess physical, role, emotional, cognitive, and social functioning. Eight symptom scales measure the prevalence and severity of cancer- or treatment-related symptoms: fatigue, pain, nausea and vomiting, loss of appetite, dyspnea, insomnia, constipation, and diarrhea. One additional item captures possible financial difficulties related to the disease or its treatment.

The BN20 module is designed to complement the EORTC-QLQ-C30 in patients with brain tumors. It consists of twenty items, which can be categorized in eleven key dimensions: future uncertainty, visual disorder, motor dysfunction, communication deficits, headache, seizures, drowsiness, alopecia, itchy skin, weakness of legs, and bladder control. Like EORTC-QLQ-C30, the BN20 utilizes a four-point Likert scale ranging from “not at all” to “very much”.

### 2.3. Data Analysis

All data were scored according to the EORTC QLQ-C30 Scoring Manual [14]. Individual items within each scale were summed to obtain a total raw score, which was then linearly transformed to a 0–100 scale where 100 represents the best QoL. Symptom items were inverted before scoring to reflect a high symptom burden in a highly scored symptom. Furthermore, summary scores (SumSc) were derived from the QLQ-C30 data according to Giesinger et al., 2016 [15], the SumSc being a mean of 13 QLQ-C30 scale scores, excluding financial impact (FI) and global quality of life (QL), with all included scores reversed so that higher values indicate better functioning or fewer symptoms. This score provides an overall measure of the patient’s quality of life, with higher scores reflecting more favorable outcomes. Missing items within a scale were handled as recommended in the EORTC manual: scale scores were computed when at least half of the items in that scale were present. Analysis was performed on all available scales for a given scale and time point.

The scores at given times in course of follow-up were then compared to the baseline scores of the same patient as described in Osoba et al. [16]. As shown by Wei et al., 2002 [17] and Ubels et al., 2015 [18], QoL after treatment improved in progression-free patients. Therefore, as previously performed in other studies [19,20], we performed a second analysis on progressing patients only and compared the outcomes in both groups.

### 2.4. Oncological Outcome Assessment

To assess the long-term outcome of proton therapy in patients with skull base Ch and ChSa, we captured the OS, PFS, LC, and freedom from distant failure (FFDF). Follow-up time was measured from the start of PT. We specifically focused on tumor control by analyzing local and distant relapse occurrences, local relapse being defined as size progression of the tumor remnant (determined using the RECIST guidelines [21], defining progressive disease as at least a 20% increase in the diameter of target lesion compared to the smallest diameter recorded since the start of treatment) or reappearance of a tumor in case of complete resection or response. Local relapses were further categorized as in-volume, marginal, or out-of-volume depending on if the majority of the recurrent tumor was located in the high (≥95% isodose), medium (20–95% isodose), or low dose (≤20% isodose) volume, respectively.

### 2.5. Toxicity Assessment

The clinical toxicity resulting from the treatment was evaluated in two distinct time frames: acute and late toxicity, capturing the adverse events occurring within the first three months following the completion of the proton therapy and beyond these three months, respectively. The toxicity assessment was conducted following the Common Terminology Criteria of Adverse Events (CTCAE). Severe toxicity was characterized as events reaching a grade 3 or higher on the CTCAE scale.

### 2.6. Statistical Analysis

Score changes compared to baseline were analyzed using descriptive statistics. To assess the correlation of clinical parameters with the observed QoL and symptom burden, correlation coefficients and *p*-values were computed using the Friedman test, *p* < 0.05 being considered statistically significant. The correlation coefficient shows how strongly two variables are related, ranging from −1 in case of a perfect negative correlation to +1 (perfect positive correlation), and 0 meaning no correlation.

Oncological outcome was assessed utilizing Kaplan–Meier survival curves, OS being determined from the start of PT up to the time point of death with censoring at loss to follow-up. Statistical analysis was performed in IBM SPSS Statistics for Windows, Version 28.0. (IBM Corp, Armonk, NY, USA).

## 3. Results

### 3.1. Patient Characteristics

A total of 77 eligible patients were included in the final clinical analysis. After excluding three patients due to missing baseline questionnaires, a total of 74 patients were included in the Osoba plot analysis. The presence of a synchronous or metachronous unrelated malignancy was not an exclusion criterion, but these events were documented and considered in the interpretation of outcomes. Patients with only one month of follow-up were also included as long as they had completed at least two QoL assessments during PT, as these data contribute meaningfully to the analysis of baseline values and capture the QoL during treatment. Figure 1 shows a CONSORT flow diagram of inclusion to the study. Thirty-one (40.3%) patients were male, and the median age at treatment start was 50.0 years (range, 18.8–79.5). The majority of patients (*n* = 48, 62.3%) were diagnosed with Ch, while 29 (37.7%) had ChSa. Compression of the brainstem or OA by the tumor was recorded in 9 (11.7%) and 22 (28.6%) cases, respectively. All patients had their disease confirmed histologically, mostly as a part of resection, with three (3.9%) cases of biopsy only. Most tumors were partially resected (*n* = 55, 71.4%), with a median residual tumor size of 8.4 cm^3^ (range, 0.3 cm^3^–96.1 cm^3^). Complete resection was achieved in 22 (28.6%) cases. Patients underwent a median of one surgery (range, 0–4) prior to PT. Chemotherapy was administered only in one patient (1.4%), who had a synchronous squamous cell carcinoma of the head and neck. The median prescribed PT dose was 74 Gy (relative biological effectiveness, RBE) (range, 68–75 Gy RBE). Table 1 details the patient characteristics. We compared our current cohort to a cohort from a previous study at our institute [3]. This comparison was carried out purely in terms of clinical outcomes, as no analysis of QoL was available for the previous cohort. Patients of the previous cohort were significantly younger and had significantly less gross total resection and larger residual tumors (Table 2). Furthermore, they exhibited a significantly higher percentage of brainstem or OA compression. The 5-year OS was 86.4% in 2016 compared to 88.8% OS in the current cohort (95% CI: 0.75–0.96%).

### 3.2. Oncological Outcome

The median follow-up time was 51 months (range 1–94). Forty-six patients (59.7%) reached the 5-year follow-up time point. Estimated OS rates were 100%, 93.1%, and 88.8% at 1, 3, and 5 years, respectively (95% CI for 5y-OS: 80.2–97.4%). Four (5.2%) patients, all of them with Ch, died as a result of tumor progression. Three other patients (3.9%) died of unrelated causes: one due to pneumonia, one due to synchronous malignancy, and one due to a metachronous glioblastoma WHO grade 4 occurring 2 years after PT. In the latter case, the tumor was localized in the low-dose area of proton radiation and developed two years after PT. Due to the time interval, a secondary malignancy is unlikely but cannot be ruled out.

PFS was 94.2%, 81.0%, and 74.0% at 1, 3, and 5 years, respectively (95% CI for 5y-PFS: 62.2–85.8%).

**Figure 2:**
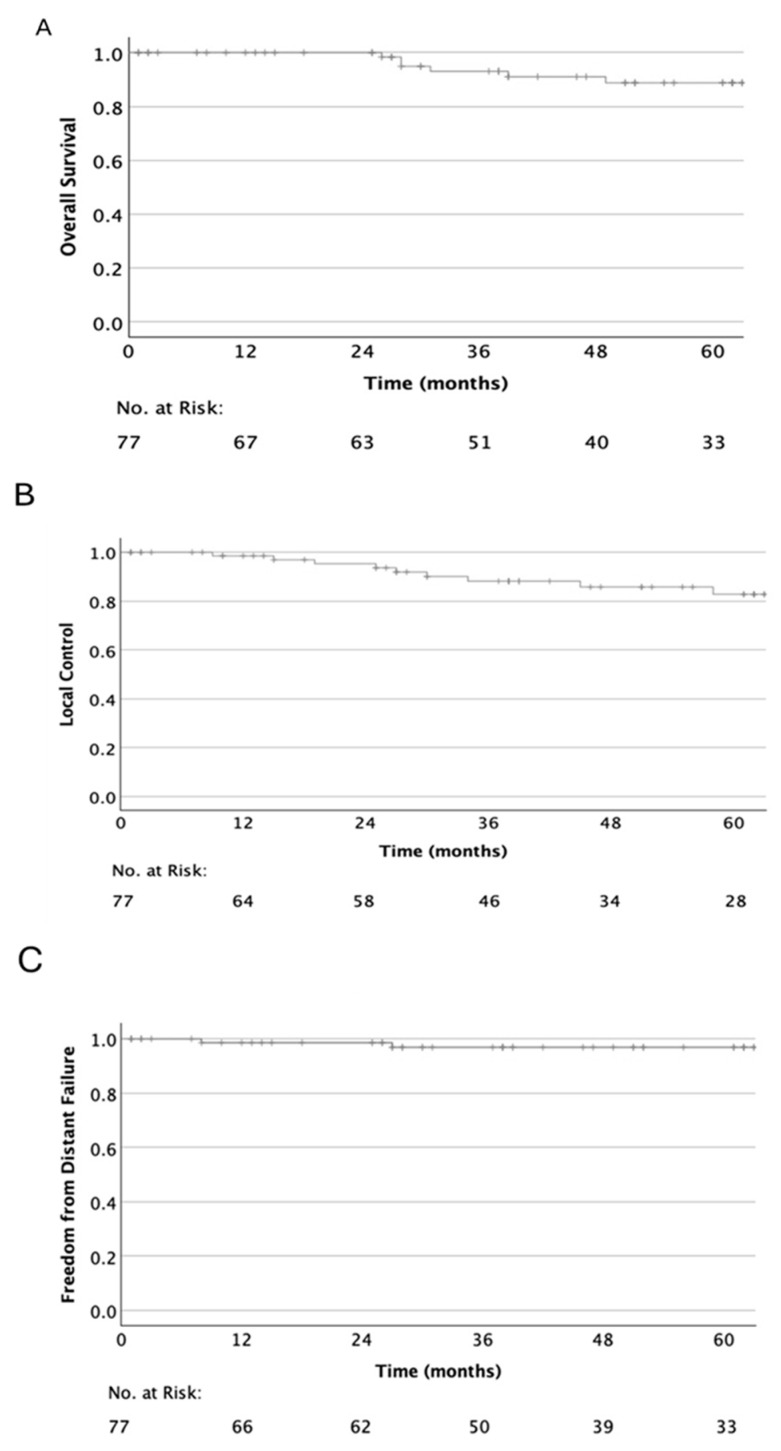
Kaplan–Meier curves of overall survival (**A**), local control (**B**), and freedom from distant failure (**C**) in 77 patients with skull base chordoma (*n* = 48) and chondrosarcoma (*n* = 29) after spot-scanning proton therapy. FFDF: 98.6%, 96.9%, and 96.9%.

LC was 98.5%, 88.2% and 82.8% at 1, 3, and 5 years, respectively (95% CI for 5y-LC: 72.2–93.4%) (Figure 2). While there was no marginal failure, ten (7.7%) patients had relapses occurring within the target volume and two (1.5%) relapsed outside of it. Distant failure occurred in two (2.6%) Ch patients in the form of leptomeningeal metastases of the cervical and lumbar spine and bone metastases, respectively. The median time to local or distant failure was 22 months.

In line with the previously published literature [3,6,7,8,9,10], the clinical outcomes of Ch and ChSa differed in favor of ChSa. The 5-year OS was 84.3% for Ch (95% CI, 72.0–91.8) and 94.7% for ChSa (95% CI, 77.9–99.0). The corresponding 5-year LC was 76.1% (95% CI, 60.0–86.5) and 93.5% (95% CI, 75.5–98.6), respectively.

### 3.3. Toxicity

In line with the known favorable safety profile of PT in skull base tumors, no grade 3 or higher acute toxicities were observed. Most (90.6%) patients experienced mild acute toxicities, out of which 54.1% were grade 1 and 35.6% grade 2. The most common acute toxicity was fatigue (*n* = 37, 52.8%), followed by alopecia (*n* = 27, 38.6%), nausea (*n* = 24, 34.3%), radiation dermatitis (*n* = 20, 28.6%), and headache (*n* = 12, 17.1%).

Late toxicities were documented in 66.3% of the patients, most of them being grade 1 (20.3%) or 2 (43.2%). The most common late toxicities were hypopituitarism (*n* = 16, 31.4%), radiation brain necrosis (*n* = 15, 29.4%), and fatigue (*n* = 11, 21.6%). Of all patients with radiation brain necrosis, eight (53.3%) were asymptomatic, and six (40%) had mild symptoms. Only two patients (2.8%) had severe late toxicities: one grade 3 brain radiation necrosis and one grade 4 optic neuropathy.

### 3.4. EORTC QLQ-C30 and QLQ-BN20 Outcome

#### 3.4.1. Response Rates

The response rate to the questionnaires decreased over the follow-up period. It was highest at both baseline and end of PT with 96.1% (74 out of 77) of responders. It decreased to 79% after 1 year (57 of 72 patients who reached this time point), 74% after 2 years (49 of 66), and 63% (38 of 60) after 3 years and remained similar at 55% (28 of 51) after 4 years, 59% (27/46) after 5 years, and 51% (18/35) after 6 years.

#### 3.4.2. C30 Global QoL

Directly at the end of PT, 50% of the patients reported worsened global QoL. Subsequently, two and three years after PT, 50% of patients exhibited a decrease in global QoL compared to baseline, while 26.2% and 25% reported improvement at these time points, respectively. After year 3, the proportion of patients reporting worse symptoms at baseline decreased, until at six years after PT, 47.1% reported improved QoL compared to baseline. The median time to treatment failure being 22 months, this points towards a substantial influence of tumor progression on reported QoL.

Examining the C30 global QoL scores specifically in patients with progressive disease (PD) (*n* = 14), congruent with the overall cohort, worsening of QoL directly after completion of PT was at 50%, decreasing to 22.2% after one year. However, two and three years after PT, a deterioration in QoL was noted in 44.4% and 57.1% of patients, respectively, which aligns with the calculated median time to failure. From the fifth year onward, a trend towards improvement in QoL reemerged, with the proportion of worsened QoL receding to 33.3%.

#### 3.4.3. C30 Sum Score

**Figure 3:**
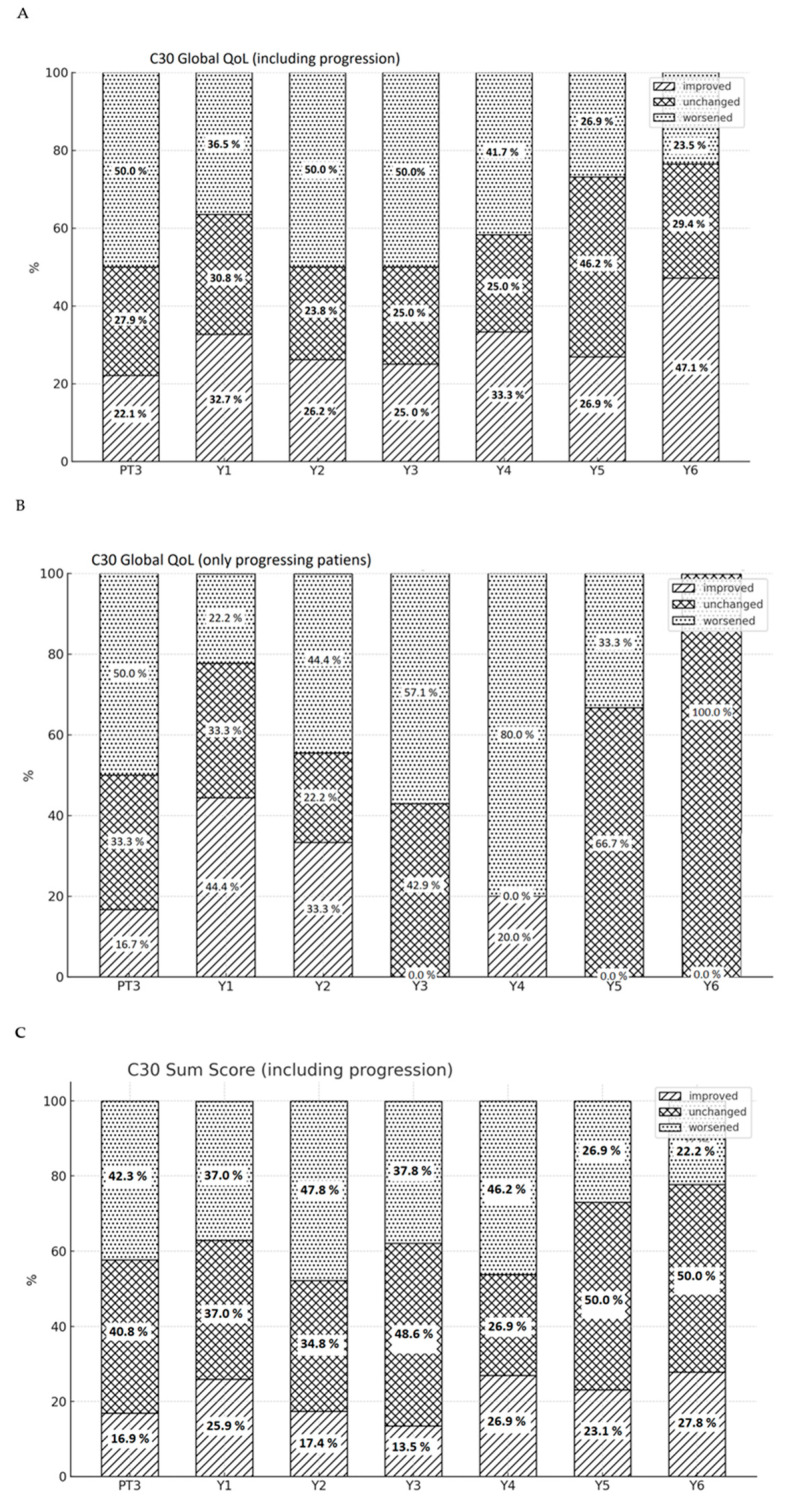
Proportions of patients reporting improved, unchanged, or worsened global QoL directly at proton therapy completion (PT3) and yearly thereafter (Y1–6) (**A**). Reported global QoL in a sub-cohort consisting exclusively of patients with progressive disease (**B**). Reported C30 SumSc in the whole cohort (**C**). The values were always compared to each patient’s baseline scores.

Like in global QoL, the analysis of C30 SumSc revealed a deterioration in 42.3% of patients directly upon PT conclusion. One year thereafter, the SumSc worsened in 37%, while in 25.9% it improved compared to baseline. Consistent to global QoL, a renewed deterioration after the second year was noted in 47.8% of patients, which then recessed to 37.8% after the third year. This trend persisted from the fifth year onward. Figure 3A–C show the corresponding Osoba plots.

#### 3.4.4. Symptom and Function Scores

Among all general symptom scores of the QLQ-C30, only fatigue and headache were consistently present directly before the initiation of PT, with 53 (71.6%) and 33 (44.6%) of patients presenting these symptoms, respectively. Fatigue symptoms persisted over time, while headaches were reported at a similar level. The rate of fatigue and headache at 3-year FU was 78.6% and 46.4%, respectively. Notably, neither of the two symptoms exhibited a significant worsening after the conclusion of PT.

Although fatigue was frequently reported at baseline, the majority of patients rated these symptoms as mild. This resulted in mean fatigue baseline score close to the general population reference level. It increased during and at completion of PT and decreased again at one year after PT, followed by further increase towards the third and fourth year. From then on, a trend towards the general population mean was observed again.

**Figure 4:**
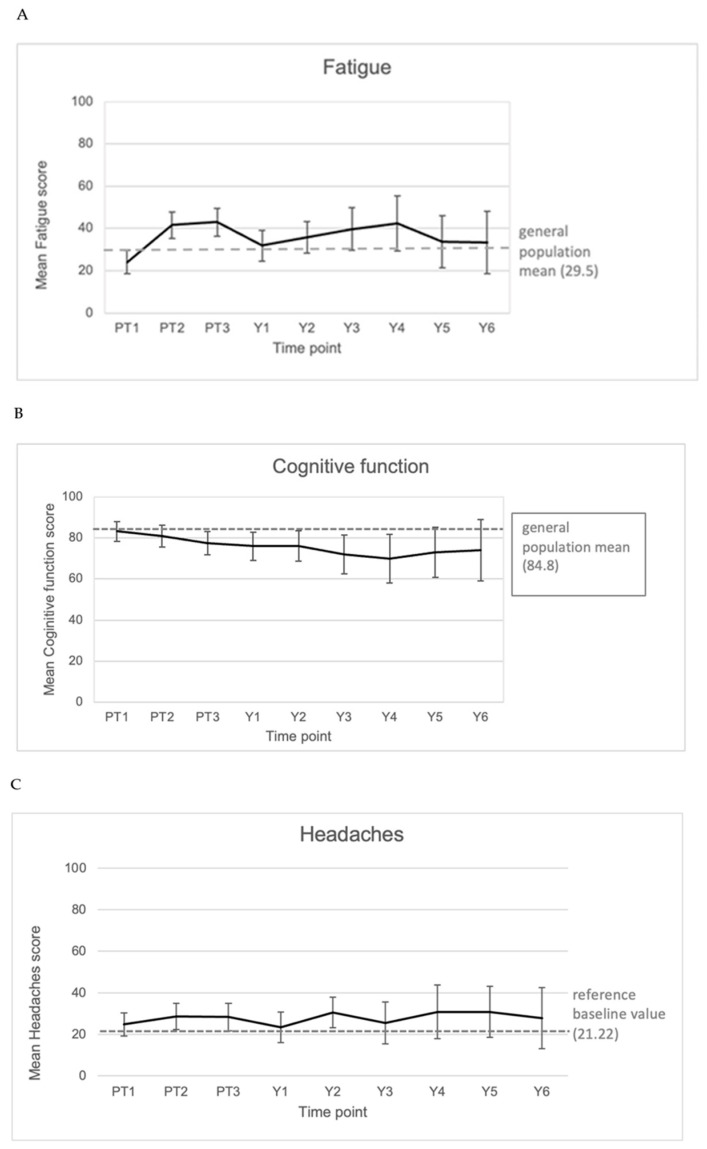
Mean values of fatigue symptoms (**A**), cognitive function score (**B**), and headaches (**C**) reported directly before (PT1), during (PT2), and immediately after (PT3) the proton therapy and annually thereafter (Y1–7) in our cohort. In dashed line, the EORTC reference value for QLQ-C30 items [22] and the EORTC reference baseline value [13] for BN20 items are displayed.

The mean cognitive function score showed a slow, gradual decrease in the course of follow-up and tended to stabilize from the fourth year post-PT. The mean was lower than the general population mean already before the start of PT (Figure 4).

The mean headache score followed a balanced course with minor fluctuations around years 2, 4, and 5 post-PT.

### 3.5. Correlation of Clinical Parameters to Reported Quality of Life

To assess the relationship between clinical and QoL outcomes, global QoL, QLQ-C30 SumSc, and QLQ-BN20 symptom scores at 1-year follow-up were compared to clinical parameters and tested for correlation. At this time point, factors associated with PT, such as radiation dose, acute toxicity, and gross tumor volume (GTV), did not show any influence on reported QoL. Nevertheless, the extent of resection exhibited a clear correlation to improved QoL throughout all time points, with the exception of C30 SumSc 1 year after the end of PT, where it trended towards the same result (*p* = 0.06; Table 3). Disease progression during the follow-up period was correlated with overall poorer quality of life already before PT, with lower global QoL and C30 SumSc and higher neurological symptom burden before PT, as well as lower C30 SumSc and neurological symptoms directly and one year thereafter. Men experienced better QoL, in terms of global QoL and C30 SumSc, and less neurological symptoms both before and one year after PT. At the conclusion of PT, there was only a difference in regard to neurological symptoms between genders, without correlation to global QoL and C30 SumSc. Table 3 and Table 4 show Friedman test correlation coefficients and *p*-values for tested clinical parameters.

## 4. Discussion

The aim of this study was to assess the clinical outcome and QoL in patients with skull base tumors treated with PT. Previous research on this topic has been limited to only one study by Srivastava et al., 2013 [7], examining a cohort of 17 patients pre- and immediately post-PT, without extended follow-up data. Our study provides long-term follow-up data of 77 patients, allowing for an analysis of the impact of PT on their QoL over time.

Our results confirm that PT is safe and effective for these tumors, with an OS of 89% and a LC of 83% at 5 years and only two patients (2.8%) having high-grade late toxicity. QoL changes throughout the course of the follow-up in this cohort, with an initial decrease directly at treatment completion, followed by an improvement thereafter. We observed a further deterioration in QoL at 2 to 3 years after PT, which aligns with the calculated time to failure of 22 months. We identified three factors significantly affecting the QoL in this cohort: resection status, post-treatment disease progression, and gender.

We found that the occurrence of post-treatment disease progression was correlated with decreased QoL both before and after PT. This may be attributed to an unfavorable tumor localization and poor prognosis, leading not only to a heightened symptom burden but also to reduced feasibility of complete resection. The significant impact of the resection status on the risk of recurrence is well known from previous research [8,9,23]. Also, previous studies at our institute [3,24] revealed a correlation of residual tumor volume to clinical outcome. Consistent with these findings, our current analysis shows a significant correlation of gross total resection with improved QoL across all time points, highlighting once again the crucial role of aggressive surgical removal in treating these neoplasms. We compared our cohort to a cohort from our previous study [3]. Patients of the previous cohort were significantly younger and had significantly less gross total resection and larger residual tumors (Table 1). Furthermore, they exhibited a significantly higher percentage of brainstem or OA compression. We noted an improvement in the rate of high-grade (>Grade 3) late toxicities: from 8.1% in 2016 to 2.8% in the current cohort. Additionally, clinical outcomes, as indicated by the 5-year OS, showed improvement, with the current cohort demonstrating an OS of 88.8% compared to 86.4% in 2016. Thus, our current findings collectively point towards a significant improvement in neurosurgical techniques and patient care, resulting in better clinical outcomes in terms of OS and recorded toxicities.

We observed fluctuations in the reported QoL over the course of time following PT. As expected, the data revealed an initial decline in QoL directly at treatment conclusion, which we primarily attribute to acute PT-related toxicities. Over the course of one year thereafter, there was a continuous improvement, suggesting a recovery period with a decrease in acute toxicities and regain of functional capacities. Interestingly, the data shows a subsequent decline in QoL at two- and three-year marks post-PT, aligning with the calculated median time to treatment failure of 22 months in our cohort. Disease recurrence or progression likely impacts not only patients’ symptom burden and functional status but also their psychological well-being, which might be leading to observed deterioration in their QoL. Another possible explanation for this decline is the decreasing questionnaire response rates over time, particularly among recurrence-free or asymptomatic patients—as it may have contributed to an overrepresentation of symptomatic individuals in follow-up assessments [25]. The trend towards improvement in QoL re-emerged from the fourth year of the follow-up period onward, indicating a possible adaptation to tumor- or treatment-related symptoms. These findings suggest an interplay between disease progression, experienced symptoms, and the perceived well-being of our patients.

There were notable gender differences in the reported QoL within our cohort. Male patients exhibited higher QoL scores compared to females, both pre- as well as one-year post-PT. This is consistent with our previous research [26], where female gender of patients with brain tumors was found to be adversely correlated with QoL one year after PT. Other studies also indicate that women report more symptoms and lower quality of life after tumor treatment compared to men, with statistically significant differences found across various cancer types [27,28,29,30]. Although the phenomenon was previously described, its underlying causes remain unclear from the available literature. Geue et al. [28] suggest a possible reporting bias, whereby women may be more likely to disclose symptoms and emotional distress. Other potential contributing factors, such as psychosocial influences or neuroendocrine effects of the tumor or its treatment, are possible but have not been thoroughly investigated in the current studies. In comparison to the general population mean, the reported mean cognitive function scores in our cohort were generally lower. The data on cognitive functioning in patients with skull base Ch and ChSa after PT is limited. Glosser et al. analyzed cognitive functioning after irradiation of seventeen patients with skull base Ch and ChSa, without evidence of adverse changes [31]. Similarly, Nugent et al. assessed cognitive functioning prior to any therapy in a cohort of patients with skull base tumors, finding no significant difference in the neuropsychological status from that of the general population [32]. However, as measured by a work limitations questionnaire in the same study, some participants reported dysfunctions in their time management skills and in meeting mental/interpersonal demands, with a consecutive alteration of their work functioning. Patients in our cohort were not routinely tested for their neuropsychological functions. Given the high impact of cognition on patients’ day-to-day functioning and overall QoL, further investigation of cognitive and neuropsychological function over time is warranted.

There were several limitations to our study. While our QoL data collection was prospective, the analysis of correlations with clinical parameters was conducted retrospectively. This approach may result in bias and limitations in interpreting the relationships between variables. Even though the EORTC QLQ-C30 and BN20 questionnaires are well validated and were scored according to their official scoring manuals [14], their aggregate scores treat all encompassed factors as equally weighted, which may not accurately reflect their relative clinical importance. This limitation is partly mitigated by reporting both a summary score and a global QoL item, as the relative weighting of items may vary between patients. As in all questionnaire-based analyses, statistically significant differences in QoL scores do not necessarily reflect clinically meaningful changes. The analysis according to Osoba et al. introduces groupings as a proxy for clinically meaningful differences, although even these cannot fully capture the individual patient’s subjective burden.

Due to the generally rare occurrence of Ch and ChSa, the sample size of our cohort is relatively small. This limits the statistical power for our analysis, which may lower the generalizability of our findings. Additionally, a multivariate analysis was not performed because of the low number of failure events relative to the study cohort. The slowly progressing nature of Ch and ChSa itself implies the necessity of an extended follow-up period to capture a sufficient amount of treatment failures or late treatment-related events necessary for a robust statistical analysis. Although the median follow-up of 51 months in our study appears reasonable compared to other published data, a study with longer follow-up is needed to fully assess the long-term effects. Another limitation of this study is the combined analysis of Ch and ChSa. Although both entities share treatment paradigms and are traditionally reported together in the literature, their biological differences could not be fully explored due to limited subgroup sizes. It is also important to consider possible response bias of the patients’ cohort. Previous research shows that participants in QoL studies may tend to feel that responding to questionnaires about symptoms no longer present is unnecessary [33]. This can result in a decreased response rate. On the contrary, as revealed by Solberg et al. in 2011 [25], patients with complications might be more likely to respond, leading to a possible over-representation of such cases and shaping of the overall assessment towards a poorer outcome.

## 5. Conclusions

PT is a well-established, safe, and effective treatment for skull base Ch and ChSa, with a favorable toxicity profile and good clinical outcomes. We identified three main factors significantly affecting QoL in this cohort: gender, resection status, and disease progression. The QoL fluctuates throughout the follow-up period, with two main declines: directly at PT conclusion and two to three years thereafter. These declines are most likely to be attributed to treatment-related acute toxicities and disease progression, respectively. Prospective multicenter studies with larger patient populations are needed to confirm these findings.

## Figures and Tables

**Table 1:** Patient characteristics (*n* = 77 patients, current study).

Category		Median (Range) or Proportion (%)
Included		77
Age [years]	Median	50.0
	Range	18.8–79.5
Sex	Male	31 (40.3%)
	Female	46 (59.7%)
Histology	Ch	48 (62.3%)
	ChSa	29 (37.7%)
Compression	Brainstem	9 (11.7%)
	Optic apparatus	22 (28.6%)
Resection status	GTR	22 (28.6%)
	Subtotal	52 (67.5%)
	Biopsy only	3 (3.9%)
GTV size [cc]	Median	8.4
	Range	0.3–96.1
PT dose [Gy (RBE)]	Median	74
	Range	68–75

Abbreviations: GTR: gross total resection, GTV: gross total volume, Gy (RBE): Gray (relative biological effectiveness), Ch: chordoma; ChSa: chondrosarcoma, PT: proton therapy.

**Table 2:** Comparison of the 2016 and current cohort patient characteristics. Significant *p*-values are highlighted in bold.

Category		Cohort 2016	Cohort 2023	*p*-Value
Total		*N* = 222	*N* = 77	
Age [years]	Mean	42.8	50.7	**0.001**
Sex [%]	Male	52.7	40.3	0.06
	Female	47.3	59.7	
Compression of any type (brainstem or optic apparatus) [%]	Yes	50.9	36.4	
	No	49.1	63.7	**0.03**
Resection status [%]	GTR	3.2	28.6	**<0.001**
	Subtotal	96.8	71.4	
GTV size [cc]	Mean	35.7	15.5	**<0.001**

Abbreviations: GTV: gross tumor volume. GTR: gross total resection.

**Table 3:** *p*-values for the tested factors in relation to C30 global QoL, C30 SumSc, and BN20 neurological symptoms before proton therapy (PT1), at the end of it (PT3), and one year after (Y1). Significant *p*-values are highlighted in bold.

Friedman Test Significance (*p*-Value)	PT1			PT3			Y1		
	Global QoL	C30_SumSc	Neurological Symptoms	Global QoL	C30_SumSc	Neurological Symptoms	Global QoL	C30_SumSc	Neurological Symptoms
Sex (male/female)	**0.011**	**0.001**	**0.027**	0.209	0.058	**0.031**	**0.008**	**0.048**	**0.015**
Age	0.473	0.286	0.821	0.526	0.946	0.338	0.844	0.881	0.664
Resection status (GTR/subtotal)	**0.003**	**0.01**	**0.007**	**0.002**	**0.001**	**0.002**	**0.034**	0.06	**0.015**
Pre-PT CNS symptoms	0.642	0.934	0.912	0.186	0.316	0.461	0.539	0.878	0.539
GTV volume	0.331	0.639	0.55	0.178	0.208	0.791	0.696	0.592	0.702
Dose	0.661	0.821	0.881	0.278	0.531	0.179	0.703	0.692	0.597
Acute toxicities	0.745	0.267	0.825	0.147	0.055	0.539	0.966	0.165	0.233
Late toxicities	0.833	0.674	0.839	0.176	0.995	0.646	0.157	0.523	0.91
PD	**0.009**	**0.013**	**0.039**	0.053	**0.02**	**0.017**	0.478	**0.038**	**0.039**

Abbreviations: CNS: central nervous system, GTR: gross total resection, GTV: gross tumor volume, PD: progressive disease, Pre-PT: before proton therapy, PT: proton therapy.

**Table 4:** Correlation coefficients between tested factors and C30 global QoL, C30 SumSc, and BN20 neurological symptoms at three time points: before (PT1), at the end of (PT3), and one year after proton therapy (Y1). Significant correlations are highlighted in bold.

Friedman Test Correlation Coefficient	PT1			PT3			Y1		
	Global QoL	C30_SumSc	Neurological Symptoms	Global QoL	C30_SumSc	Neurological Symptoms	Global QoL	C30_SumSc	Neurological Symptoms
Sex (male/female)	**0.296**	**0.369**	**−0.257**	0.148	0.222	**−0.251**	**0.345**	**0.263**	**−0.322**
Age	−0.085	−0.126	0.027	−0.075	−0.008	0.113	−0.027	0.02	0.059
Resection status (GTR/subtotal)	**0.34**	**0.372**	**−0.311**	**0.358**	**0.366**	**−0.347**	**0.281**	0.251	**−0.321**
Pre-PT CNS symptoms	0.055	0.01	−0.013	0.155	0.118	−0.087	0.083	−0.021	−0.083
GTV volume	−0.137	−0.067	0.085	−0.188	−0.176	0.037	0.06	0.082	−0.059
Dose	0.052	0.027	−0.018	−0.128	−0.074	0.158	0.052	0.054	0.072
Acute toxicities	0.039	−0.131	0.026	−0.17	−0.224	0.073	−0.006	−0.187	0.161
Late toxicities	0.025	−0.05	−0.025	0.159	0.001	−0.054	0.19	0.086	−0.015
PD	**−0.301**	**−0.28**	**0.241**	−0.226	**−0.269**	**0.277**	**−0.096**	**−0.275**	**0.274**

Abbreviations: CNS: central nervous system, GTR: gross total resection, GTV: gross tumor volume, PD: progressive disease, Pre-PT: before proton therapy, PT: proton therapy.

## Data Availability

The data will be made available by the authors upon reasonable request.
